# Dissecting the complex regulation of lodging resistance in *Brassica napus*

**DOI:** 10.1007/s11032-018-0781-6

**Published:** 2018-02-21

**Authors:** Charlotte N. Miller, Andrea L. Harper, Martin Trick, Nikolaus Wellner, Peter Werner, Keith W. Waldron, Ian Bancroft

**Affiliations:** 1 John Innes Centre, Norwich Research Park, Norwich, NR4 7UH UK; 2 Present Address: Department of Biology, University of York, York, YO10 5DD UK; 3 Quadram Institute Bioscience, Norwich Research Park, Norwich, NR4 7UA UK; 4 KWS UK Ltd., 56 Church Street, Thriplow, Hertfordshire, SG8 7RE UK

**Keywords:** Lodging, Associative Transcriptomics, Stem strength, Pectin demethylesterification, Marker-assisted breeding

## Abstract

Lodging continues to be a major cause of yield loss in important crop species such as *Brassica napus.* Understanding the genetic regulation of lodging resistance is therefore of key interest to breeders worldwide. Current strategies aimed at minimising lodging risk involve the incorporation of dwarfing genes or the application of plant growth regulators. However, despite these efforts, lodging continues to be a persistent problem and it is therefore of high interest that novel, complimentary strategies for lodging control are implemented. One approach would be to focus on understanding the genetic properties underlying stem mechanical strength. With this in mind, we screened a training genetic diversity panel of *B*. *napus* accession for variation in stem mechanical strength and related traits. Using Associative Transcriptomics, we identified molecular markers for a suite of valuable traits. Using an independent test genetic diversity panel, we show that the methods employed are robust for identification of predictive markers. Furthermore, based on conserved synteny with *Arabidopsis thaliana*, we are able to provide a biological context to the marker associations detected and provide evidence for a role in pectin methylesterification in contributing to stem mechanical strength in *Brassicaceae.*

**Electronic supplementary material:**

The online version of this article (10.1007/s11032-018-0781-6) contains supplementary material, which is available to authorized users.

## Introduction

Oilseed rape (*Brassica napus L.*) is a crop of growing economic importance and is second only to soybean as a commercial oilseed crop. In 2014, oilseed rape contributed 71 million metric tonnes to global oilseed production (FAOSTAT). In addition to providing a high-value oil for human consumption, oilseed rape also provides a source of protein meal which is used widely as animal feed. Oilseed rape is widely used in rotation with cereal crops, aiding the management of weeds, pests and diseases. Finally, more recently, oilseed rape has been recognised as a potential feedstock for biofuel production (Petersson et al. 2007; Stephenson et al. 2008). Given the importance of this oil crop, maximising oilseed rape yields remains a key breeding aim.

One factor known to contribute significantly to yield losses in this species is lodging—defined as the permanent displacement of the crop from its usual vertical growth habit. Lodging may result from root anchorage failure, known as root lodging, or may be the result of stem breakage, also known as brackling. Both stem and root lodging are known to be influenced by environmental, agronomic and genetic factors. The reduced yields resulting from lodging are caused primarily by the uneven penetration of light through the canopy—with the upper pods of a lodged canopy receiving too much light and the lower pods receiving too little. This not only reduces overall seed yield but also significantly reduces seed quality. In addition to hindering light interception, a lodged canopy also causes complications for mechanical harvesting, making the process more time-consuming. It is estimated that in the UK, up to 35% of oilseed rape grown is affected by lodging, leading to an estimated loss of up to £214/ha (Kendall et al. 2012). There is therefore great value in the implementation of methods aimed at reducing lodging risk.

Previous efforts to reduce the occurrence of lodging in oilseed rape have focused on reducing the height of plants through the application of plant growth regulators (PGRs). The two commonly used PGRs for lodging control of oilseed rape are metconazole and tebuconazole (Berry and Spink 2009). Given the difficulty in predicting lodging risk, these chemicals are often applied as a precaution, which in years where lodging is not a significant problem, leads to unnecessary costs. Furthermore, continued pressure to minimise treatment of food crops with chemicals is likely to result in reduced acceptability of metconazole and tebuconazole for use in lodging control. In addition to the use of PGRs, some progress has also been made in the introduction of dwarfing genes for improved lodging resistance. The introduction of the *Brassica rapa*, *Brrga1-d* allele into a *Brassica napus* (*B. napus*) background resulted in a significant height reduction and decreased lodging risk. However, additional pleotropic effects of this allele, including a delay in flowering time, may make the incorporation of this allele undesirable under certain growth conditions (Muangprom et al. 2006). An alternative strategy would be to breed varieties with increased stem mechanical strength. While stem mechanical strength is recognised as an important agronomic trait, very little research has been focused towards the identification of markers that could be used in breeding programs with the aim of reducing lodging susceptibility in oilseed rape. Furthermore, the few mapping studies that have been conducted with this aim have taken a QTL analysis approach which is limited by relatively low mapping resolution due to the limited number of generations over which recombination has occurred (Udall et al. 2006).

Genome-wide association scans, or GWAS, promises to overcome the inherent limitations seen with QTL analysis by making use of historical recombination events which, when coupled with high marker density, promote an unparalleled increase in mapping resolution. In recent years, we have seen the successful application of GWAS in a number of different plant species (Atwell et al. 2010; Hwang et al. 2014; Miller et al. 2016; Pasam et al. 2012). Furthermore, important contributions made by Harper et al. (2012) have illustrated the power of combining variation at both the gene sequence and gene expression level in a novel method termed, Associative Transcriptomics (Harper et al. 2012). In a proof of concept study, this method proved powerful in dissecting the genetic control of both seed erucic acid and seed glucosinolate content (Harper et al. 2012)—both important traits in the breeding of oilseed rape. More recently, this method has been used successfully in elucidating the genetic control of anion homeostasis in *B*. *napus* (Koprivova et al. 2014).

Our aim was to explore the available variation for lodging-related traits across a panel of *B*. *napus* accessions and, using Associative Transcriptomics, identify molecular markers that can be used for the selection of this variation in future breeding programs and start to explore the potential molecular basis of the variation observed (i.e. to look for plausible candidate genes). We show that Associative Transcriptomics is a powerful approach for identification of molecular markers that have potential for use in marker-assisted breeding for increased stem strength. Furthermore, by making use of the genomic resources available in the closely related species, *Arabidopsis thaliana*, we provide evidence of an important role of cell wall pectin demethylesterification in contributing to variation in stem mechanical strength in *Brassicaceae.*

## Methods

### *B*. *napus* plant material and phenotyping

Plant material was harvested across 3 years. The first 2 years (grown in 2010 and 2011) consisted of small panels of *B*. *napus* accessions grown at KWS UK Ltd., Thriplow (with field location varying between years). At both sites, accessions were grown in single, randomised, 1-m^2^, open-field plots. Forty-six *B*. *napus* accessions were grown in 2010 (K2010 trial; Map Reference TL402464; latitude: 52.098447; longitude: 0.046606064; silty loam soil; approximate plant spacing 7 cm) and 44 accessions in 2011 (K2011 trial; Map Reference TL398453; Latitude: 52.088450; Longitude: 0.039610863; medium loam soil; approximate plant spacing 7 cm). In each year, where possible, ten plants were harvested from each accession. In 2012, a larger trial consisting of 79 *B*. *napus* accessions was conducted under field conditions at The John Innes Centre (J2012 trial; map reference: TG 182075; Latitude: 52.62138; Longitude: 1.22217; sandy loam soil; approximate plant spacing 30 cm). This trial consisted of plants grown in three randomised blocks. For each accession, one plant was grown in each block. These plants were grown within a net-enclosed cage to reduce damage caused by pigeons, which were a problem on this site. Supplementary Data File 1 provides an overview of the accessions included in each trial. For all trials, harvesting was carried out by hand using secateurs. Plants were harvested 10 cm from the base of the stem (to reflect typical combine cut heights). Following harvest, in some cases, damage to the stem caused by handling of the plants during harvest was observed. All stems for which such damage was noticed were omitted from all further analyses. All harvested plants were dried at room temperature prior to further processing.

**Fig. 1:**
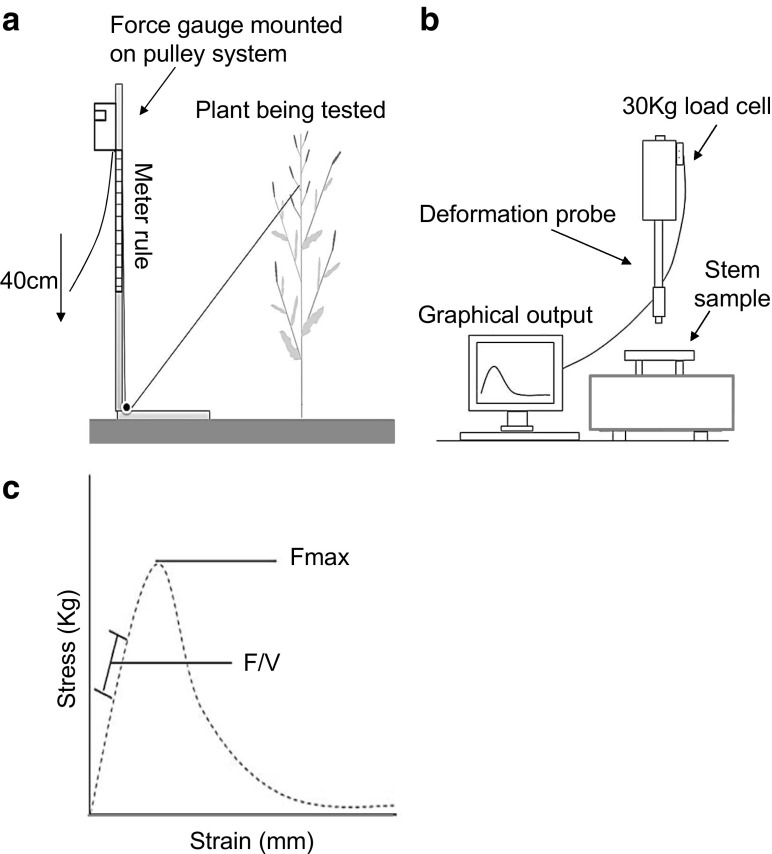
Methods use for assessment of stem mechanical strength in *Brassica napus.* A field-based measure of stem lodging risk was implemented using a pulley stem equipped with digital force gauge (**a**). To complement this, a lab-based 3-point bend test was also used (**b**). This provides a real-time graphical output of stem deformation, from which the stem absolute strength traits Fmax and *F*/*V* can be extrapolated (**c**)

Prior to harvest, all plants grown in trial J2012 were screened for lodging risk. This was carried out at maturity just prior to harvest using a digital force gauge mounted on a pulley system (as illustrated in Fig. 1a). Attached to the stem just below the fourth branch (from the top of the plant) using fishing wire and a metal clamp, the amount of force required to pull the plant 40 cm from its upright growth habit was determined directly from the force gauge reading. No lodging had been observed in this field prior to assessment. The use of the pulley system allowed plants to travel through a reproducible arc path similar to the movements induced by heavy wind or rainfall. This experiment was performed across all plant replicates and a mean stem lodging risk (SLR) score calculated for each genotype.

To allow for an in-depth analysis of the relationship between plant morphology and stem strength/lodging risk, plant height and stem weight were scored across all genotypes. Following this, a 20-cm section was taken from the base of the stem. To obtain stem cross-sectional measurements (required for the later calculation of stem second moment of area (*I*) and collection of other morphological data), the transverse of the most basal stem end was photographed. All images were later analysed using the digital analysis software Sigma Scan (Stystat Software Inc., San Jose, USA), allowing the following cross-sectional measurements to be determined: whole stem area (used in the later calculation of *D2*), stem hollow area (defined as hollow owing to a lack of or very reduced parenchyma tissue in the central cavity of the stem) (used in the later calculation of *d1a*), the area of stem parenchyma (or pith) and the thickness of the stem outer cortex.

To compensate for variation in moisture content, following the collection of cross-sectional information, all samples were further dried in a silica chamber. To assess the length of time required for the moisture content of the stem samples to equilibrate with that of the silica chamber, a pilot study was carried out. This involved the storage of samples in an air-tight silica chamber at a constant temperature of 23 °C and recording any daily changes to stem sample weight. This study showed that after 6 days, no further change in stem weight was observed suggesting that sample moisture content had equilibrated with the silica chamber conditions (data not shown). For this reason, prior to mechanical testing, all stem samples were dried for a minimum of 6 days at 55% RH and 23 °C in the silica chamber.

To screen for stem mechanical strength in *B*. *napus*, a three-point bend test method was used. This screen was carried out on the lower portion of the stems of each plant (following the equilibration of stem moisture content with the previously described silica chamber) and was performed using the Texture Analyser (TA) (Analyser (TA-XT2®-Stable Microsystems, Godalming, UK) with a three-point bend test setup (Institute of Food Research, Norwich, UK) (Fig. 1b). These methods and calculations were adapted from Kern et al. (Kern et al. 2005). The TA was fitted with a load cell with maximum loading capacity of 30 kg. The support stands were set at 7 cm apart (across which the 20-cm stem sample was placed) and the testing probe was set to move at a constant speed of 2 mm/s. The TA, connected to a computer, produces a real-time graphical output, representing the mechanical profile of the stem sample being tested. From this graph, Fmax, the absolute resistance of the stem sample to break under load, and *F*/*V*, the resistance of the stem sample to bend elastically, were obtained (Fig. 1c). These are ‘absolute strength measures’, being the result of a combination of both strength due to structure and material strength. These absolute measures of strength, together with the stem sample second moment of area (I) (Eq. 1), were used in calculating the material strength of the stem samples: the modulus of rupture (MOR), describing the resistance of the stem material to break under load (Eq. 3) and the modulus of elasticity (MOE) describing the resistance of the stem material to bend elastically (Eq. 2).

### Equations

#### Equation 1

##### The second moment of area

It is a measure of the geometrical properties of, in this case, a hollow beam which summarises the distribution of material around the central axis.

1 $$ I=\pi \left(\mathrm{D}24-\mathrm{D}1\mathrm{a}4\right)/64 $$

where

D2: diameter of whole stem calculated from stem cross-sectional area
D1a: diameter of stem hollow calculated from stem hollow area

#### Equation 2

##### Modulus of rupture

The modulus of rupture describes the resistance of the stem material to breakage under load.

2 $$ \mathrm{MOR}=\left(\mathrm{Fmax}\ast \mathrm{a}\ast \mathrm{D}2\right)/I $$

#### Equation 3

##### MOE

MOE describes the flexural stiffness of the stem material under load.

3 $$ \mathrm{MOE}=\left(F/V\right)\ast \left(\mathrm{a}2/12\right)\ast \left(3\mathrm{L}-4\mathrm{a}\right)/I $$

where

*L*: the length of the stem sample between the two supports
*a*: L/2

### Statistical analysis

To assess trait normality and trait variance components, analysis of variance (ANOVA) was performed using Genstat 15th edition. Where non-normality was observed, the raw trait values were transformed using a log_10_ transformation and the ANOVA repeated using transformed data values. To determine whether the accessions varied significantly for the various traits, a minimum significance level of *P* < 0.05 was implemented. Following the calculation of trait means, Genstat was used to undertake regression analysis.

### Associative Transcriptomics: mRNAseq and marker detection in *B*. *napus*

All accessions included in this study were sequenced using Illumina transcriptome sequencing. To allow for the identification of SNPs between these accessions, the sequencing reads were aligned to a reference sequence comprised of a collection of 94,558 unigenes. These unigenes were assembled using publicly available ESTs. Using a high-density linkage map (Bancroft et al. 2011) together with *B*. *rapa* (Wang et al. 2011) and *B*. *oleracea* genome scaffolds (Liu et al. 2014), these unigenes were assembled into a hypothetical gene order. The ordering of these unigenes was also aided by information regarding conserved synteny between Arabidopsis and *B*. *napus*. These assembled sequences, or pseudomolecules (see Supplementary Data File 2 for pseudomolecule V4—a revised version of the *B*. *napus* pseudomolecule published by Harper et al. (2012)), were then used in place of a full reference genome sequence. Alignment of the mRNAseq data to this reference sequence (using MAQ, as described in Harper et al. 2012) identified 225,001 SNP markers. In addition, quantification of sequence read depths (as reads per kb per million aligned reads; RPKM) provided a measure of expression for each unigene. RPKM values therefore provide the information required for exploring the relationship between gene expression and the trait of interest in what has been termed a gene expression marker analysis, or GEM analysis. A full description of the methods used for the detection of these markers can be found in Trick et al. (2009) and Harper et al. (2012).

### Associative Transcriptomics: accounting for population structure and relatedness and performing Associative Transcriptomics in *B*. *napus*

To assess the level of broad-scale population ancestry, STRUCTURE analysis was used (Falush et al. 2003). This method allows the number of populations (K) between which the genome ancestry of the accessions can be apportioned to be determined. Following this analysis and further calculations as described by Evanno et al. (2005) (Evanno et al. 2005), the best estimate of K was revealed to be 2. Supplementary Data File 3 provides a plot of these two populations as generated by STRUCTURE and gives a summary of the calculations used in estimating K. Based on these results, a Q matrix, describing the apportioning of the *B*. *napus* accessions between the two detected populations, was constructed.

### Associative Transcriptomics: association analysis

To undertake association analysis, the constructed Q matrix was used in mixed linear model (MLM) analysis in TASSEL (Bradbury et al. 2007). The SNP data file available (see Supplementary Data File 4) consisted of 225,001 SNP markers. Following the removal of minor alleles present at < 5%, this data set was reduced to 144,131 SNPs. This filtered SNP data set was used in TASSEL to create a Kinship matrix and as marker input for the consequent Associative Transcriptomics analysis. The GEM analysis was carried out as described previously (Harper et al. 2012). This analysis utilised transcript abundance of 189,116 unigene sequences (the transcript abundance data used for the GEM analysis can be found in Supplementary Data Files 5 and 6 for the A and C genomes respectively) (Harper et al. 2012). All highly associating GEM markers were further analysed by mapping their respective RPKM values as a trait against the SNP data.

### Growth and phenotyping Arabidopsis T-DNA insertion lines

T-DNA insertion lines were obtained from the Nottingham Arabidopsis Stock Centre (NASC). Supplementary Data File 7 provides a list of the T-DNA mutants analysed. Seeds were grown in P40 trays (H.S.P ltd vacapak, UK) containing Scotts Levington F2 mix (Everris, The Netherlands). Following stratification at 5 °C for 2 days, all plants were grown in a controlled environment growth room (22 °C, 75% RH and 1 h day). When seedlings were successfully established, leaf material was harvested in liquid nitrogen for DNA extraction.

To allow for extraction of genomic DNA, leaf samples were ground to a fine powder and 200 μl of TE extraction buffer added (200 mM TrisHCL pH 7.5; 250 mM NaCl; 25 mM EDTA and 0.5% SDS). Following further mixing, samples were centrifuged at maximum speed for 3 min. The resulting supernatant was then added to 150 μl of 100% isopropanol. Following thorough mixing by inverting, samples were centrifuged for 7 min at maximum speed. The resulting pellet was then washed with 70% ethanol and resuspended in 40-μl distilled water. Genotyping of T-DNA mutants was carried out using primers listed in Supplementary Data File 6 and using Takara Ex taq (Clontech-Takara Bio Europe) as per manufacturer instructions. All PCRs were performed using a G-storm-GS1 thermocycler. Primer design was carried out using the Salk Institute Genomics Laboratory (SIGnal) T-DNA primer design tool: http://signal.salk.edu/tdnaprimers.2.html.

Following the identification of homozygotes, mutants were grown for a second generation for phenotyping. After 4 weeks of growth, plants were transferred to ARRASYSTEM trays with supportive tubing (Beta Tech bvba), allowing for the plants to bolt and mature with no need for staking.

At maturity, plants were harvested and prepared for mechanical testing as follows. A 2.5-cm section was taken from the base of the stem using a scalpel. This was carried out, where possible, from six plants per genotype. This section was then stored in a small breathable bag and stored within a silica humidity tank at 55% RH and 23 °C for 2 days. The cross-section of the lower stem transverse was photographed to allow for stem morphological measurements to be collected as described previously for *B*. *napus*. To accommodate the much smaller stem samples, a support stand was made using two razor blades (blade covered and pointing downwards) held within a clamp. These supports were set to be 1.2 cm apart. The Texture Analyser was fitted with a 1-kg load cell. The probe was set at a start height of 4 cm from the test bed and the probe was set to descend a further 0.5 cm following contact with the stem sample.

### FTIR analysis of T-DNA mutant stem tissue

Fourier transform infrared (FTIR) spectra were collected across the spectral range of 800–4000 cm^−1^ using a dynamic alignment FTIR spectrophotometer (Bio-Rad FTS 175C, Bio-Rad Laboratories, Cambridge, USA). Prior to analysis of stem tissue, a spectrum of air was generated which acted as the reference medium. Each stem sample was macerated using a pestle and mortar and the ground tissue clamped against a diamond element. To reduce error, the crystal was completely covered with stem tissue. Between samples, the crystal and clamp were cleaned with 100% ethanol. This was left to dry thoroughly before analysing the next sample. For each sample, two spectra were taken, with three plant replicates per genotype analysed. All spectra obtained were truncated to the fingerprint region (800–1800 cm^−1^). The baseline was anchored to 1800 cm^−1^ and the curve areas normalised. Average spectra were then obtained for the mutant line and the values subtracted from those of WT Arabidopsis. This allowed for any difference in cell wall composition of the T-DNA insertion line relative to WT to be detected.

### Marker validation

Eighty-six accessions of *B*. *napus* (genotype unknown) were harvested from a test panel grown at Nottingham University. These genotypes formed a subset of the Renewable Industrial Products from Rapeseed (RIPR) diversity panel (Havlickova et al. 2017). All plants were grown in individual pots in a polytunnel using a completely randomised design. For each accession, where possible, three plants were harvested. However, in some cases, only one or two plant replicates were available. All plants were harvested as described for the Associative Transcriptomics accessions. DNA was extracted using the method previously described for Arabidopsis. Genome-specific primers were designed for the marker locus analysed. Assays were first tested on *B*. *napus* accessions of known genotype (a subset of the Associative Transcriptomics panel). Following confirmation that the designed primers were able to effectively screen for the target variation, they were further used to genotype the 86-accession test panel. All genotyping was performed using AMPLITAQ Gold polymerase (Life Technologies Ltd. (Invitrogen Division, Paisley, UK)). Prior to sequencing, PCR reactions were purified using the ExoSAP protocol **(**Etchevers 2007). Following this, sequencing reactions were set up in 0.2-ml tubes according to a revised protocol from BigDye V3.1 terminator cycle sequencing kit (Applied.Biosystems 2002). All PCR and sequencing reactions were performed using a GStorm GS1 thermal cycler (Somerton, UK). Capillary sequencing was performed by GATC Biotech AG, Germany, and all sequencing trace files obtained were analysed using Contig Express (Vector NTI advance® 11.5.2, Paisley, UK). Following genotyping, all accessions were mechanically tested using a three-point bend test as described previously. Using a *t* test (Genstat 15th edition), the trait data and genotype data obtained were assessed for any significant marker-trait segregation patterns. Supplementary Data File 8 provides details of the validation marker assay used and Supplementary Data File 9 provides a list of the test panel genotypes included in this study.

## Results

### Variation in lodging-related traits across a panel of 79 *B*. *napus* accessions

We first assessed lodging resistance across the diversity panel. Using ANOVA, the trait data obtained from the largest trial (J2012) was assessed for normality. In all cases, approximately normally distributed residuals were observed. Through ANOVA, for all traits included in the study, we observed high levels of phenotypic variation between genotypes (*P* = < 0.01), as might be expected for an association panel of diverse accessions.

Two smaller trials were conducted, K2010 and K2011. In the majority of cases, accessions were replicated across both trials. However, a number of accessions were represented in just a single year (see Supplementary Data File 1). Due to the unbalance nature of these data sets, the data obtained from each field trial were analysed independently. Through ANOVA of the 2010 and 2011 trial results, with the exception of stem hollow area in the K2010 analysis (*P* = 0.618), significant differences were seen between accessions for all of the traits assessed (*P* < 0.05). Data summaries and all ANOVA outputs for each of these field trials can be found in Supplementary Data File 9. Given the more extensive variation represented by the J2012 trial, this data set was used for the majority of analyses discussed in the following sections. However, the data obtained from K2010 and K2011 provide validation of trends observed in the J2012 data set and will, for this reason, be discussed where appropriate.

### Relationships between stem mechanical strength and stem morphological and structural traits

To assess the importance of stem morphological and structural traits on stem mechanical strength, regression analysis was conducted. A summary of the results obtained from this analysis is given in Table 1. Many significant correlations were observed. Firstly, the absolute strength traits Fmax and *F*/*V* correlate positively with an *R*^2^ of 0.88 (*P* < 0.001). This suggests that these traits are highly related, i.e. stems resistant to bending are also more resistant to breaking. These absolute strength traits also correlate significantly with a number of stem morphological and structural traits. The structural strength trait, second moment of area, correlates positively with both Fmax and *F*/*V* with respective *R*^2^ values of 0.63 (*P* < 0.001) and 0.66 (*P* < 0.001). Stem diameter was found to explain a greater level of variation than second moment of area in these absolute strength traits with an *R*^2^ value of 0.70 (*P* < 0.001) for both Fmax and *F*/*V*. Stem outer cortex thickness and parenchyma area were also found to correlate positively with Fmax and *F*/*V*. These traits are also highly related to stem diameter and second moment of area. It is therefore difficult to assess whether these tissues themselves are contributing to strength, or whether the correlations are due to their relationship with a more general increase in stem thickness.

**Table 1:** Pearson’s correlation coefficient (tested against zero) for traits measured across *Brassica napus* panel artefacts

	FSV	Fmax	MOE	MOR	Stem_diameter	Second_moment of area	Parenchyma_area	Outer_cortex_thickness	Hollow_area	Plant_height	Stem_weight
Fmax	***0.88										
MOE	***− 0.23	***− 0.25									
MOR	**− 0.13	**− 0.11	***0.84								
Stem_diameter	***0.69	***0.69	***− 0.57	***− 0.52							
Second_moment_of_area	***0.66	***0.61	***− 0.29	***− 0.31	***0.87						
Parenchyma_area	***0.52	***0.54	***− 0.46	***− 0.42	***0.80	***0.64					
Outer_cortex_thickness	***0.46	***0.43	***− 0.25	***− 0.18	***0.47	***0.46	***0.16				
Hollow_area	***0.09	**0.13	*− 0.05	**− 0.09	***0.22	***0.29	*0.07	*0.05			
Plant_height	***0.35	***0.39	***− 0.14	**− 0.11	***0.39	***0.35	***0.28	***0.23	***0.19		
Stem_weight	***0.63	***0.61	***− 0.17	*− 0.11	***0.53	***0.58	***0.37	***0.31	***0.18	***0.23	
Mean_SLR_score	***0.33	***0.42	***− 0.21	***− 0.16	***0.42	***0.33	***0.31	***0.17	***0.14	***0.25	***0.26

***Indicates significance at *P* ≤ 0.001 and **indicates significance at *P* ≤ 0.01 and *indicates significance at *P* ≤ 0.05

A strong relationship was detected between the absolute strength traits and stem weight where *R*^2^ values of 0.62 (*P* < 0.001) and 0.64 (*P* < 0.001) were seen for Fmax and *F*/*V* respectively. When assessing the relationships between this biomass trait and the stem structural and morphological traits, we observed that the correlations with Fmax and *F*/*V* were stronger. Similar correlations were also identified between stem weight and stem diameter/second moment of area. It is therefore likely that the relationship observed between stem weight and absolute strength reflects this, but may also suggest that stem density is important. Stem absolute strength was also seen to correlate positively with plant height, with *R*^2^ values of 0.35 and 0.39 obtained for *F*/*V* and Fmax respectively.

Interestingly, when looking at the relationship between stem absolute and stem material strength traits, a negative relationship can be seen. Stem material strength describes the mechanical strength contributed by the material composition of the stem and is considered independent of stem structural strength which is described by the stem geometry. Of course, these strength components are not truly mutually exclusive, but it is useful, when aiming to understand the underlying genetics, to break down complex traits into component traits—in this case material strength, structural strength and absolute strength (material and structural strength combined). Fmax and *F*/*V* are used in calculating MOR and MOE respectively. Based on the equations used, it would be expected that an increase in these absolute strength traits would result in increased material strength values. However, when looking more closely at the raw data, it can be seen that in the majority of cases, high absolute strength values can be largely explained by high structural strength (large diameter/high second moment of area) and in these accessions, low material strength is often observed. While this suggests that stem structural strength is the most important contributor to absolute stem strength in these accessions, it does not mean that the material strength properties do not have a role to play. When looking at the mean trait values, there are several examples which illustrate a certain importance of material strength in determining overall stem strength. An example can be seen when comparing the trait data for the varieties, Dwarf Essex and Taisetu. The absolute strength values for Dwarf Essex are 24.2 and 19.9 kg/s for Fmax and *F*/*V* respectively. The mean values for stem diameter and second moment of area for this accession are 9.2 and 362 mm^4^ respectively. When comparing these data to those obtained for Taisetu, it can be seen that despite displaying very similar values for stem diameter and second moment of area (9.288 and 365.8 mm^4^ respectively), Taisetu shows much lower absolute strength values of 6.259 and 8.79 kg/s for Fmax and *F*/*V* respectively. This may be explained by the much lower material strength seen for this accession in comparison to that of Dwarf Essex. For Taisetu, MOR and MOE values of 2.52 and 173.4 N/mm^−2^ were seen. In comparison, Dwarf Essex was found to have a mean MOR of 11.03 N/mm^−2^ and a mean MOE of 39.1 N/mm^−2^. This clearly shows the effect that material strength can have in the absence of variation in stem structural strength. As seen in the regression analysis results carried out for the 2012 JIC trial material, in the analyses carried out on material from the 2010 and 2011 KWS trials, the absolute strength traits Fmax and *F*/*V* were found to correlate most significantly with stem diameter, second moment of area and stem weight (data not shown).

### The relationship between stem lodging risk and stem mechanical strength

**Fig. 2:**
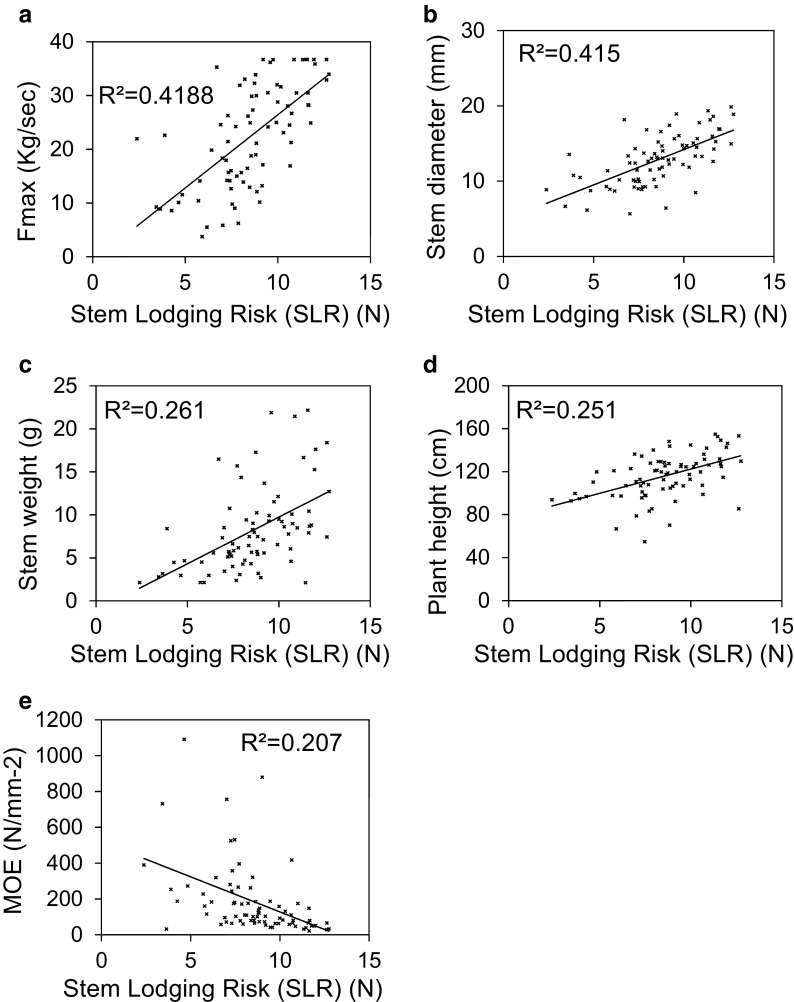
The relationship between stem lodging risk and stem absolute strength, structural and morphological traits. Significant positive correlations were observed between the field-based stem lodging risk assay and the lab-based 3-point bend test measure of stem absolute strength (**a**) and stem diameter (**b**). Stem lodging risk also correlates positively with stem weight and plant height (**c**, **d**). Stem lodging risk correlates negatively with the material strength trait, MOR (**e**)

Using the apparatus illustrated in Fig. 1a, the J2012 material was assessed for stem lodging risk (SLR) under field conditions. Assessment of the data obtained through ANOVA revealed significant variation for this lodging risk trait between accessions (*P* = 0.043). Through a further regression analysis, the relationship between this field-based scoring and the mechanical strength traits obtained from the three-point bend test were explored. Some of the key relationships observed are plotted in Fig. 2. Significant positive correlations were observed between the absolute strength traits and the SLR scores measured in the field with *R*^2^ values of 0.42 (*P* < 0.001) and 0.33 (*P* < 0.001) for Fmax (Fig. 2a) and *F*/*V* respectively. These lodging risk scores were however found to correlate negatively with the material strength measures with *R*^2^ values of 0.16 (*P* < 0.001) for MOR and 0.21 (*P* < 0.001) for MOE (Fig. 2e) respectively. This again illustrates the importance of stem structural strength in determining stem strength in *B*. *napus*. Indeed, very similar correlations were observed between the stem structural traits stem diameter and second moment of area and the SLR score as those seen for absolute strength. For both of these structural traits, positive correlations were observed with *R*^2^ values of 0.42 (*P* < 0.001) and 0.37 (*P* = 0.001) for stem diameter (Fig. 2b) and second moment of area respectively. Stem height and plant weight were also found to correlate positively with the SLR scores (Fig. 2c, d) with respective *R*^2^ values of 0.25 (*P* < 0.001) and 0.26 (*P* < 0.001). However, given that these correlations are weaker than those seen between SLR and the absolute strength measures, it seems that the variation in these morphological phenotypes has not had a confounding effect on the SLR measurements. The detection of these significant correlations is very promising and suggests that the mechanical strength measures obtained through the three-point bend test are capturing important variation relevant to stem mechanical strength under field conditions. Furthermore, as with the results obtained from the three-point bend test assay, the results presented here suggest that understanding the genetic control of stem absolute strength and stem structural strength (with contributions made by stem diameter and stem second moment of area being of highest value) would be of great interest for the improvement of lodging resistance in *B*. *napus.* The work presented also confirms the widely accepted link between plant height and lodging susceptibility.

### Associative Transcriptomics for stem absolute and structural strength traits, Fmax, structural and morphological traits

In order to identify molecular markers that might be useful to support breeding for traits of relevance for lodging resistance, we conducted association analysis by Associative Transcriptomics.

Through regression analysis, we showed that stem lodging risk is most highly related to the absolute stem strength traits Fmax and *F*/*V*. Given the very high correlation observed between Fmax and *F*/*V*, we limited the consequent Associative Transcriptomics analysis to Fmax only. We also explored marker-trait associations for several stem structural traits that showed high correlation with absolute stem strength. The structural traits included were stem diameter, stem second moment of area, stem parenchyma area, outer cortex thickness and stem weight. Finally, given the known role that height has to play in contributing to lodging risk (which was confirmed in the present study), variation in plant height was also included in the Associative Transcriptomics analysis. An overview of the most highly significant SNP and GEM marker associations identified for each trait is provided in Supplementary Data File 10. Full Manhattan plots for all traits analysed can be found in Supplementary Figures 1–9. With the exception of one marker (JCVI_19156:300), the highly associated markers occur at similar frequencies in the sub-populations defined by STRUCTURE, as shown in Supplementary Figure 10, confirming their association with trait variation is not simply due to population structure within the panel.

**Fig. 3:**
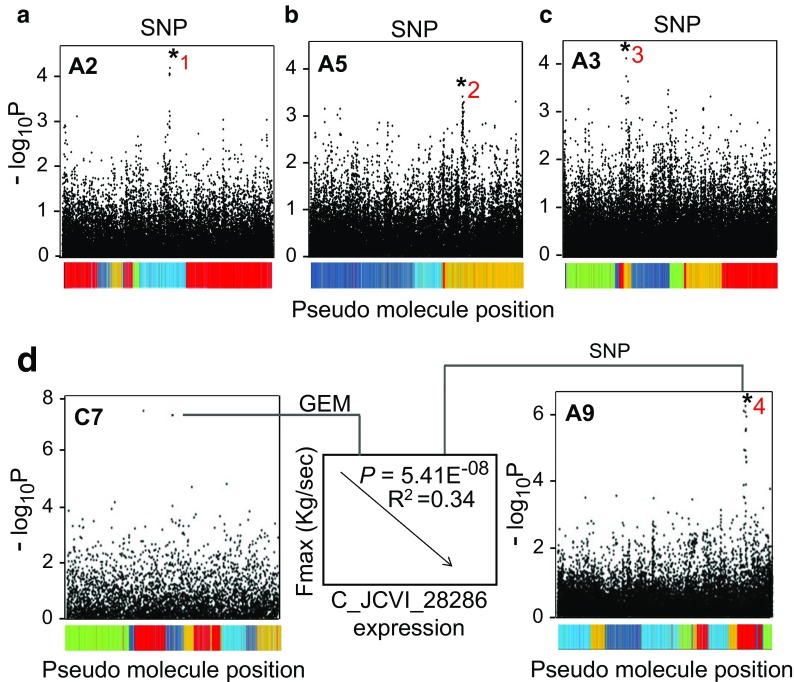
Associative Transcriptomics reveals loci underlying the regulation of the absolute stem strength trait, Fmax. Three SNP associations located on the homoeologous regions of chromosome A2/C2 (marked as *1) (**a**) A5/C5 (marked as *2) (**b**) and A3/C3 (marked as *3) (**c**) were identified in the Associative Transcriptomics analysis carried out for the absolute strength trait, Fmax. In each case, just a single plot for the associating A genome homoeologue is shown here. Highly significant GEM associations were also identified for this trait. When mapped as a trait in a further SNP analysis, variation in transcript abundance for one associating marker, C_JCVI_28286, located on chromosome C7 (**d**), maps to variation at several genomic loci including chromosome A9 (marked as *4) (**d**), suggesting the presence of a genetic interaction between these loci. These marker associations were based on data obtained from J2012. The coloured bars along the *X* axis represent the linkage groups of the *B*. *napus* pseudomolecule with each colour denoting conserved synteny between *B*. *napus* and *Arabidopsis thaliana* where light blue, orange, dark blue, green and red denote *A*. *thaliana* chromosomes 1, 2, 3, 4 and 5, respectively

For the absolute strength trait, Fmax, three main SNP association peaks were identified based on the results obtained from J2012 trial (a full Manhattan plot for these data can be found in Supplementary Figure 1). The first and most significant of these was found to be on chromosome A2/C2 (the A2 association can be seen marked with *1 in Fig. 3a). The most significant marker within this peak was JCVI_31359:1657 (*P* = 4.45E-05, trait effect = 30.9% (a percentage based on trait range across accessions)). This region was also detected (more weakly) in the SNP association analysis carried out for the K2010 material for absolute stem strength (Supplementary Figure 2). Identification of a common signal across two field trials is very promising. These markers were found to be in close proximity to orthologs of Arabidopsis genes expected to have pectin methylesterase (PME)/pectin methylesterase inhibitor (PMEI) activity, AT5G50030 and AT5G50060. Previous studies have found that the methylesterification state of cell wall pectin has a key role to play in determining stem mechanical strength (Hongo et al. 2012). Pectin methylesterification is also known to effect cell expansion (Wolf et al. 2009), a process which likely contributes to variation in stem thickness. As we have seen, stem diameter explains a high level of variation in stem absolute strength in *B*. *napus*, making these genes very good candidates for controlling the trait variation observed.

The second SNP association for this absolute strength trait was detected on chromosome A3/C3 (A3 association can be seen marked with an *3 in Fig. 3c) with the most significant marker being JCVI_38663:382 (*P* = 4.16E-05). No clear candidate genes were identified in close proximity to this marker.

A final SNP association for Fmax was seen on chromosomes A5/C5, with the top marker being JCVI_39914:1128 (the A5 association is shown with *2 on Fig. 3b). This marker was assigned a *P* value of 4.07E-04 and a trait effect of 26.9%. Within close proximity to these markers is again an orthologue of an Arabidopsis gene described as having PMEI/PME activity (AT3G12880). Weak signals in this region were also identified for stem weight in the Associative Transcriptomics analysis carried out on both the J2012 and K2011 trials (Supplementary Figures 3 and 4).

In addition to these very promising SNP associations, highly significant GEM associations were also detected for Fmax for the J2012 trial. A summary of these associations is given in Supplementary Data File 10. One of these GEMs, JCVI_28286, detected on chromosome C7, corresponds to an orthologue of an Arabidopsis *CYTOCHROME P450* (CYP450) (AT4G27710)*.* The expression of this gene correlates negatively with Fmax (*P* = 5.41E-08, *R*^2^ = 0.34) (Fig. 3d). In Arabidopsis, the expression of this gene has been linked with localised cell wall deposition (McCurdy et al. 2008). To further explore the role of this gene in contributing to stem mechanical strength in *B*. *napus*, the respective transcript abundance values for this marker were mapped as a trait against the SNP data. This analysis revealed SNP associations. The clearest of these associations was identified on chromosome A9/C8 (the A9 association can be seen marked as *4 in Fig. 3d). A second clear peak was identified on A6/C6. The most significant marker detected for the A6/C6 association was JCVI_11271:1070 (*P* = 1.09E-05). The second association, seen on A9/C8, had a much stronger signal, with the most significant marker being JCVI_7691:268 (*P* = 5.44E-07). In close proximity to the SNP association on A6/C6, there are several genes involved in cell wall biosynthesis. The first of these is an orthologue of Arabidopsis *INCURVATA 4* (AT1G52150). This gene is predicted to act as a transcriptional regulator, important for vascular development and cell differentiation. In addition, two genes known to contribute to cell wall mannan structure were identified. Firstly, there is a gene orthologous to Arabidopsis *MANNAN SYNTHESIS-RELATED 2* (AT1G51630). Cell wall mannans are thought to bind to cellulose, providing structural integrity to the cell wall (Rodríguez-Gacio et al. 2012). As a second example, an orthologue of Arabidopsis *ALPHA MANNOSIDASE 1* was identified. Mannosidases are a group of enzymes, involved in the cleavage of linkages between cell wall mannose and other cell wall polysaccharides (Mast and Moremen 2006). No clear candidate genes were identified in close proximity to JCVI_7691:268. These associations suggest that there may be an interaction between these loci and the CYP450 gene detected in the GEM analysis. Based on the candidate genes identified in the A6/C6 region and the expected role of this CYP450 gene in contributing to cell wall biogenesis, these findings may be indicative of a cell wall biosynthesis pathway which contributes to stem mechanical strength in *B*. *napus.*

As seen for Fmax, a clear SNP association can be seen on chromosome A2/C2 for stem diameter with the most significant marker being JCVI_31359:1651 (Supplementary Figure 5). This association signal was also identified for stem parenchyma area (Supplementary Figure 7). Although these associations are less clear than the SNP signal identified at this locus for Fmax, the detection of common associations between these traits suggests that there is a genetic component underlying the observed trait correlations. A second SNP association for stem parenchyma was seen on chromosome A1/C1 with the most significant marker, JCVI_36764:445, reaching a *P* value of 0.00023. No clear candidate genes for this trait were identified in this region.

For stem outer cortex thickness, a SNP association was seen on chromosome C6 with the most significant marker being JCVI_335:451 with a *P* value of 0.00027. Again, no clear candidate genes for this trait were found in close proximity to this marker (Supplementary Figure 8).

One clear association signal was detected for plant height. This was seen on chromosome A3/C7 where the most significant marker is JCVI_26003:352 (*P* = 2.86E-04, trait effect 21.7%) (Supplementary Figure 9). This marker is in close proximity to a gene orthologous to Arabidopsis *BRI1* (*BRASSINOSTEROID-INSENSITIVE 1*) *SUPPRESSOR 1* (AT4G30610). *BRI1* is known to be involved in regulation of plant height in Arabidopsis. Mutants defective for this gene have a very clear dwarfing phenotype (Noguchi et al. 1999). The detection of a suppressor of this gene for plant height in *B*. *napus* is therefore very encouraging. A more modest peak was also detected for plant height on chromosome A9/C8. The most significant marker is JCVI_19156:300 (*P* = 8.82E-04, trait effect of 16.8%). No clear candidate genes were detected in this region.

The GEM analysis identified two highly associating GEM markers for plant height on chromosome A5, both of which correspond to a gene orthologous to Arabidopsis *MICROTUBULE ORGANISATION 1* (*MOR1*) (AT2G35630). Arabidopsis mutants for this gene exhibit a temperature-dependant reduction in organ size. This was found to be due to the inability of the mutant plant to correctly organise cortical microtubules (Whittington et al. 2001). To further explore the potential role of this gene in contributing to the genetic control of plant height in *B*. *napus*, transcript abundance levels for the most highly associated of these GEM markers, A_EE440437 (*P* = 2.98E-07, *R*^2^ = 0.26 (correlating negatively with plant height)), were mapped as a trait against the SNP data. In doing this, a very clear SNP association on chromosome A2/C2 was uncovered. The most highly associated marker within this peak was JCVI_20133:246. No clear candidate genes were identified within this region. However, the results presented here suggest that there may be a gene, which resides at this A2/C2 locus, which either directly or indirectly regulates the expression of *MOR1*, contributing to the observed variation in plant height in *B*. *napus.*

### Validation of markers for the selection of high stem absolute and structural strength

The discovery of marker-trait associations through methods such as Associative Transcriptomics has the potential to contribute greatly to crop improvement through marker-assisted breeding. The durability of such markers can however depend on many factors including environmental interactions. It is also possible that the associations may be the result of false-positive errors due for example to any unaccounted-for population structure or relatedness between individuals used within the study. It is therefore important that the efficacy of detected markers in selecting for the trait of interest is validated. To do this, a test panel (i.e. a set of unrelated accessions not used for the original association analysis) of *B. napus* accessions of previously unknown genotype was screened for marker variation detected through Associative Transcriptomics. Following mechanical testing, it was then possible to assess whether the allelic variation segregates with the target trait as would be expected based on the Associative Transcriptomics results.

**Fig. 4:**
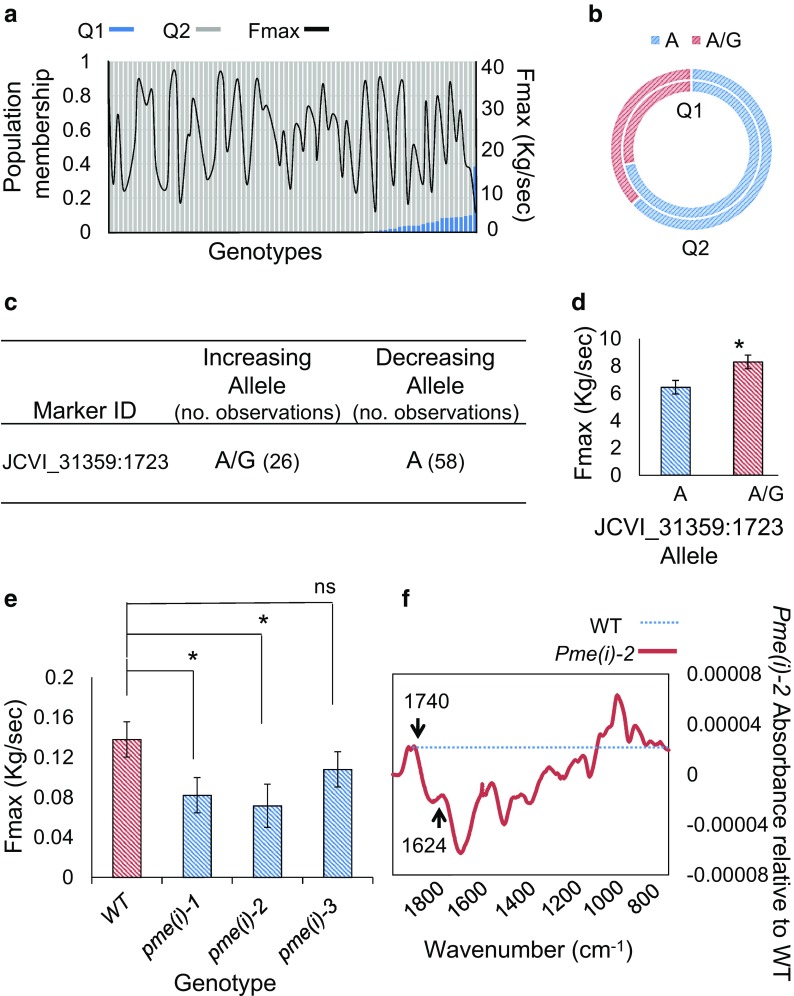
Validation of a predictive marker for Fmax. The variation in Fmax across the population shows no relationship with the sub-populations identified through STRUCTURE analysis (**a**). Allele frequencies for predictive marker JCVI_31359:1723 are approximately equal in the sub-populations (**b**). The population contains many individuals with each of the increasing and decreasing alleles of JCVI_31359:1723 (**c**). Accessions in the test panel scored as containing the increasing allele of JCVI_31359:1723 exhibit greater Fmax (*indicates *P* < 0.05) (**d**). Arabidopsis lines with knock-out mutations in *PME(I)* exhibit reduced Fmax (*indicates *P* < 0.05) (**e**). FTIR spectra for Arabidopsis *pme(i)-2* exhibit depletion at 1624 cm^−1^ and enrichment at 1740 cm^−1^ relative to WT which indicates reduced pectin demethylesterification in mutant stem tissue (**f**)

Given the importance of the absolute strength trait, Fmax, in contributing to stem lodging resistance, we focused this marker validation study on the marker association detected on chromosome A2/C2 with the most significant marker being JCVI_31359:1657. This marker was not only found to show association with Fmax across multiple field trials (across different years and environments), but was also seen for additional traits which were found to be related to stem absolute strength through regression analysis. Due to low sequence read-depth in the region surrounding the most significant marker in this association peak, an alternative, tightly linked marker, JCVI_31359:1723, was used (*P* value 9.32E-05; trait effect of 26.5%). Following the assessment of the efficacy of the developed genome-specific marker assay in screening for the target variation (using a subset of Associative Transcriptomics accessions of known genotype), the marker assay was used to explore variation across the 86-accession test panel. High levels of variation in Fmax are seen across accessions and there is no discernible relationship with the sub-populations identified through STRUCTURE analysis, as shown in Fig. 4a. The alleles scored, as summarised in Fig. 4c, had approximately equal frequencies in the sub-populations, as shown in Fig. 4b. There is a significant (*P* < 0.05) increase in Fmax associated with the increasing (A/G) allele, as shown in Fig. 4d. Together, these results showed that the allelic variation does not simply reflect population structure and that this marker can be used for selection of genotypes with greater stem strength (Supplementary Data File 7 summarises the results obtained).

### PMEI positively regulates stem strength in Arabidopsis T-DNA mutants

In addition to molecular markers to assist breeding, the Associative Transcriptomics approach is also remarkably effective at identifying candidate genes for the control of trait variation. We therefore assessed the plausibility of a candidate gene for the control of Fmax based on functional analysis of orthologues in the related species *A*. *thaliana*.

Given that two independent SNP associations identified for Fmax (and several related structural traits) were in close proximity to genes implicated in pectin methylesterification, we decided to explore the potential causality of these genes through screening Arabidopsis T-DNA mutants. Although no T-DNA insertion mutants were available for the PME/PMEI candidate genes identified on chromosome A2/C2, several mutants were available for the PME/PMEI candidate identified on chromosomes A5/C5. These mutants each carried an insertion affecting the coding regions of Arabidopsis gene model AT3G12880. Supplementary Data File 6 summarises the mutants screened and provides information regarding the primers used in determining their genotype. For simplicity, these mutant lines have been renamed as *pme(i)1* to *pme(i)3.*

Following genotyping, homozygous mutants were grown alongside wild-type (WT) control plants and the mature stem tissue mechanically tested using a three-point bend test method. Figure 4e summarises the mean Fmax values obtained for the three T-DNA lines included relative to WT plants. Two of the three T-DNA lines assessed for altered stem strength showed a significant difference in Fmax relative to WT plants. In both cases, a decrease in stem strength was observed, suggesting that this PME(I) acts to positively regulate stem strength in WT plants.

Based on information available from the Arabidopsis Information Resource (TAIR), it is not clear whether AT3G12880 has a role in promoting or inhibiting pectin methylesterification. Studies have reported high similarities in the sequence of proteins performing these seemingly antagonistic functions (Pelloux et al. 2007; Wang et al. 2013). Given this, based on sequence information alone, it is difficult to propose the mode of gene function here, i.e. whether it has a role in cleaving methyl ester pectin side groups (making it a PME), or whether it is an inhibitor of this process (making it a PMEI). To further assess the role of this gene in contributing to stem mechanical strength, FTIR analysis was carried out on the stem material used in mechanical testing for homozygous *pme(i)-2* homozygous and WT plants. Figure 4f summarises the results obtained. Reports comparing the FTIR spectra of high and low methylesterified pectin suggest that it is a shift in the ratio of two spectral peaks which is the important discriminating factor. The first of these peaks is found at 1740 cm^−1^. The second is a peak at between 1600 and 1630 cm^−1^, which would show a higher or lower absorbance relative to the 1740 cm^−1^ in low methylesterified and high methylesterified pectins respectively (Szymanska-Chargot and Zdunek 2013). As can be seen from Fig. 4f, the T-DNA mutant stems show enrichment relative to WT at 1740 cm^−1^ and depletion at 1624 cm^−1^. This suggests that this pectin-related gene is functioning as a pectin methylesterase and that a lack of demethylesterification in these mutant lines is contributing negatively to absolute stem strength.

## Discussion

Despite great breeding efforts, yield losses resulting from high lodging susceptibility continue to be a key challenge for oilseed rape breeders worldwide. The work presented here provides insights into a new approach to improving lodging resistance: increasing stem strength through increased stem thickness. Importantly, the loci identified for stem mechanical strength and plant height are independent. This provides breeders with the opportunity to select phenotypes in a combination that best suits their breeding aims. For example, while short plants with strong narrow stems may be beneficial if seed is the only crop product, greater straw biomass would be beneficial if rapeseed is to be grown as a dual-purpose crop, with straw exploited as a biomass feedstock for lignocellulosic ethanol production.

Through our analyses, we observed that much of the variation in absolute stem strength observed across our *B*. *napus* genotypes could be explained by variation in stem diameter. Although this trait is easy to score under field conditions, it is also influenced by environmental conditions and can only be assessed towards the end of the season. It would therefore be particularly valuable to have molecular markers available to support marker-assisted breeding. Through Associative Transcriptomics, we have identified a suite of markers for both absolute stem strength and structural stem strength phenotypes. To assess the efficacy of these marker in selecting for these important traits, we undertook a marker validation study. This analysis revealed that JCVI_31359:1723, which was detected across multiple years and environments as well as for multiple, related traits, proved robust for the selection of accession with high stem absolute strength. In light of the marker validation experiment, it may be advantageous to re-evaluate which of the marker associations detected through Associative Transcriptomics may hold most promise for crop improvement. The SNP marker, JCVI_31359:1723, which proved durable for the selection of Fmax in an independent panel of genotypes, obtained a *P* value of 9.32E-05. It may be appropriate to introduce a retrospective significance threshold level of 9.23E-05. All SNP associations found to reach/exceed this level of significance can be seen marked with an asterisk in Supplementary Data File 10. Based on the results obtained in the marker validation study, our conclusion is that these markers may hold most value for the improvement of these traits through marker-assisted breeding.

The results obtained from the Associative Transcriptomics analysis of Fmax and the consequent mechanical testing and FTIR analysis of Arabidopsis mutant stems implicated demethylesterification (the removal of methyl ester groups) of pectins as having an important role in determining stem strength in *B*. *napus.* This is consistent with the previous work that showed a clear role of *PME35* in regulating stem mechanical strength in Arabidopsis (Hongo et al. 2012). Pectins, together with cellulose and hemicelluloses, form an important component of the plant cell wall. Pectins are secreted into the plant wall in a highly methylesterified form. They can then be modified by enzymes such as PMEs, which are able to cleave the methyl ester group from their side chain (Xiao and Anderson 2013). There are two opposing hypotheses for the effect of pectin demethylesterification on the cell wall. One proposed effect is that of cell wall loosening resulting from the removal of methyl ester groups from the cell wall pectins. In this scenario, it is thought that the reduced methylesterification promotes the degradation of the cell wall pectin by enzymes, and that this degradation promotes cell wall loosening. The second scenario proposed suggests that the removal of methyl esters by PMEs increases the likelihood of calcium bridges forming, which ultimately rigidifies the plant cell wall (Hongo et al. 2012; Müller et al. 2013). In both scenarios, one can imagine a way through which an increase in stem strength can be achieved. In the first scenario, we see loosening of the cell wall following the increased exposure to cell wall degrading enzymes due to the absence of methyl ester groups. Although this may be interpreted as having a weakening effect on the cell wall and consequently the stem tissue as a whole, cell wall loosening resulting from the act of PMEs has also been implicated in cell expansion. For a cell to expand, the cell wall must first loosen, allowing for the incorporation of additional cell wall material (McCann et al. 2001). It is plausible that the increased cell wall loosening, resulting from high PME activity in a subset of *Brassica* accessions, is positively contributing to cell growth and consequently regulating stem thickness and stem absolute strength. It is however also possible that the strength decrease seen in the Arabidopsis T-DNA mutants results from a lack of calcium bridges forming due to the high presence of methyl ester groups.

The work presented here illustrates the power of Associative Transcriptomics for understanding the genetic regulation of complex traits in a polypoid crop species of global economic importance. The careful selection of the *B*. *napus* genotypes included in the panel has enabled the detection of high levels of genetic variation and high mapping resolution from a relatively small Associative Transcriptomics panel. We have also illustrated the importance of carrying out marker validation experiments to reveal the potential of markers identified through association analysis for the selection of traits of agronomic importance. Such confirmation provides greater confidence in the usefulness of the markers identified, strengthening the translation of scientific discovery from the lab into the field.

## Electronic supplementary material

ESM 1 (XLS 41 kb).

ESM 2 (XLS 23241 kb).

ESM 3 (XLS 326 kb).

ESM 4 (TXT 46.4 mb).

ESM 5 (TXT 62.3 mb).

ESM 6 (TXT 62.7 mb).

ESM 7 (XLS 15 kb).

ESM 8 (PDF 2556 kb).
