# Supplementary material for: Dissecting the complex regulation of lodging resistance in Brassica napus
Source: Mol Breed. 2018 Feb 21;38(3):30. doi: 10.1007/s11032-018-0781-6 (PMC5842258; doi:10.1007/s11032-018-0781-6)
Supplement: Supplementary file 8 — (PDF 2556 kb). [file 11032_2018_781_MOESM8_ESM.pdf]

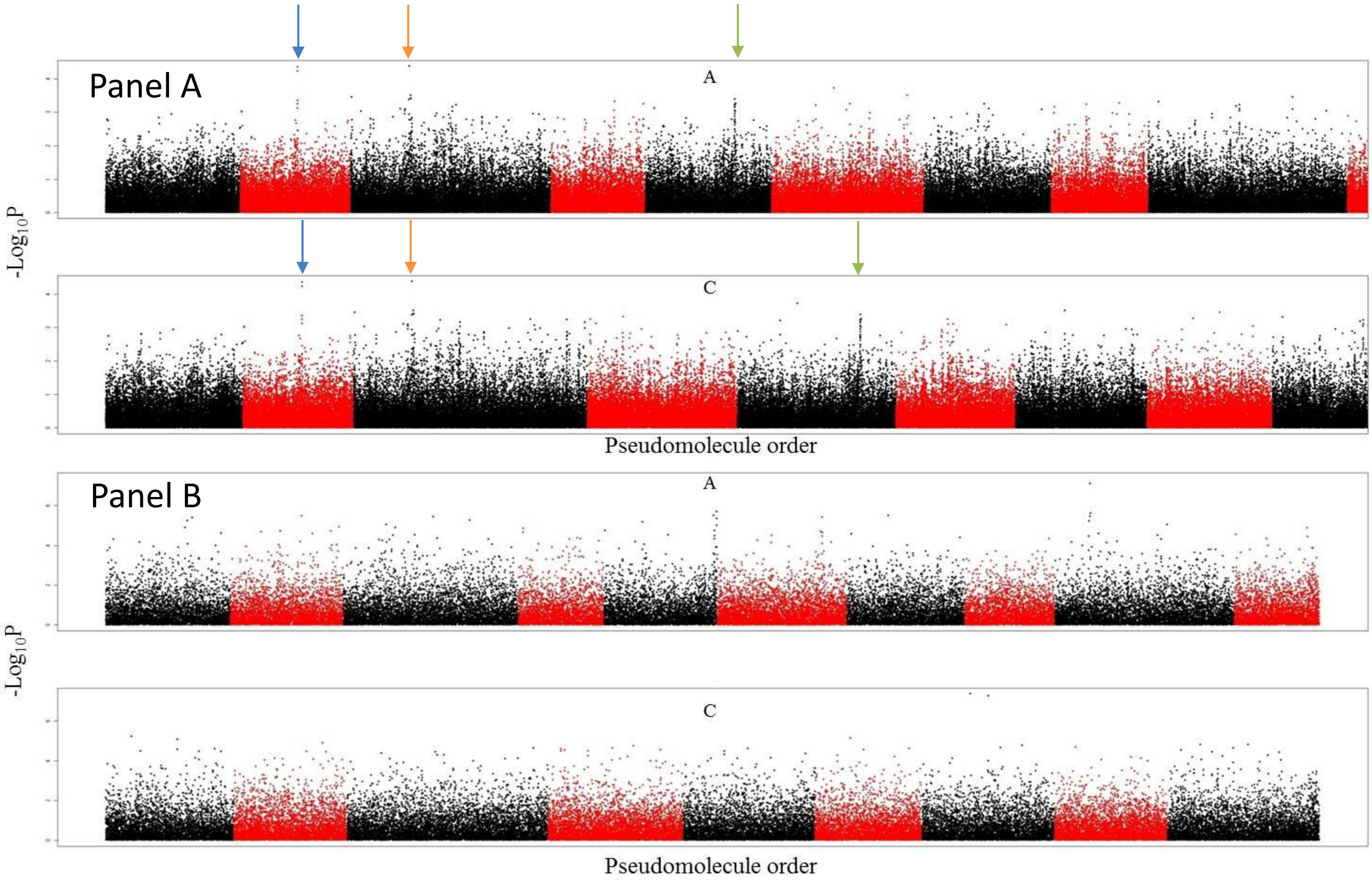

Supplementary Figure 1. Associative Transcriptomics SNP (A) and GEM (B) results for Fmax for JIC-grown *B. napus.* Significance of marker associations is shown as –Log10P and markers are plotted in pseudomolecule order. The two *B. napus* genomes can be seen marked as A and C.

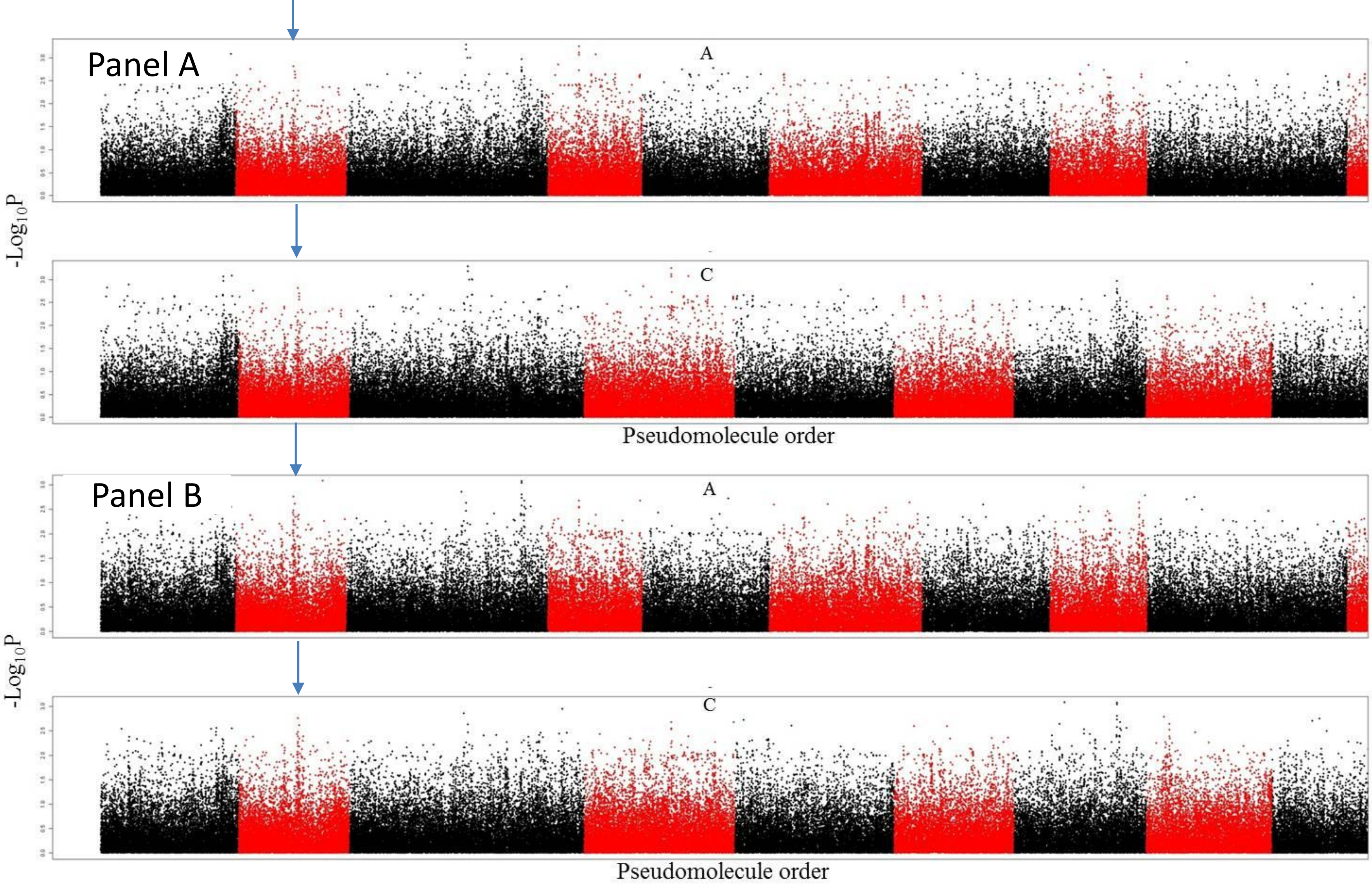

Supplementary Figure 2. Manhattan plots showing the Associative Transcriptomics SNP results for Fmax (A) and F/V (B) for KWS-2010-grown *B. napus.* Significance of marker associations is shown as –Log10P and markers are plotted in pseudomolecule order. The two *B. napus* genomes can be seen marked as A and C.

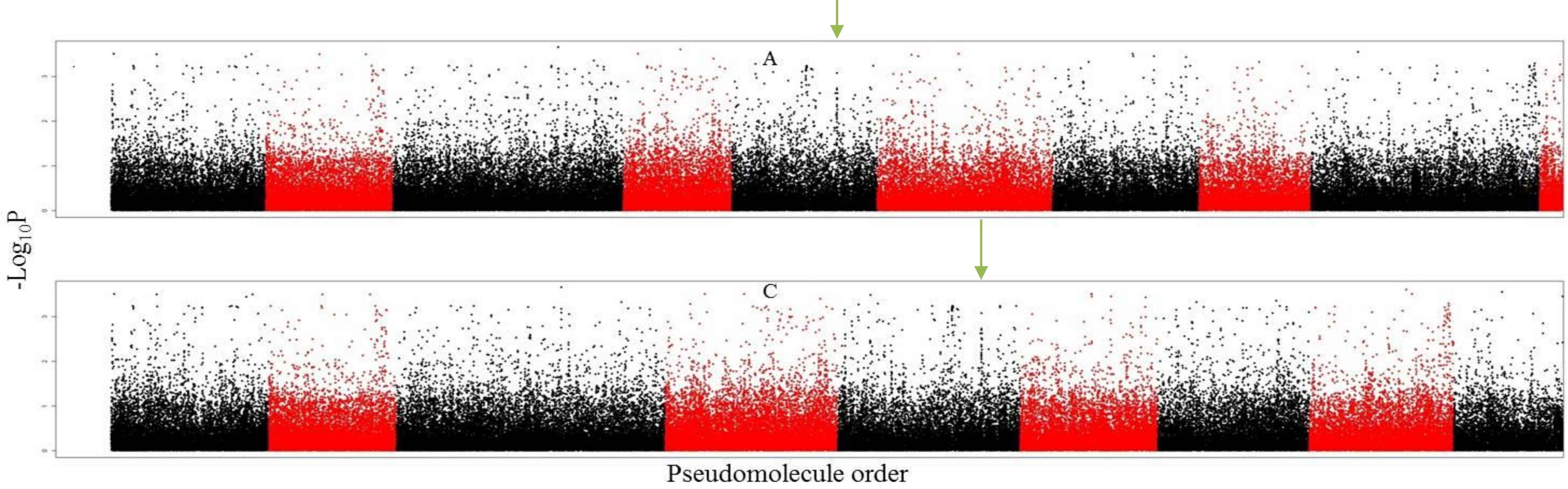

Supplementary Figure 3. Manhattan plots showing the Associative Transcriptomics SNP results for stem weight for KWS-2011-grown *B. napus.* Significance of marker associations is shown as –Log10P and markers are plotted in pseudomolecule order. The two *B. napus* genomes can be seen marked as A and C.

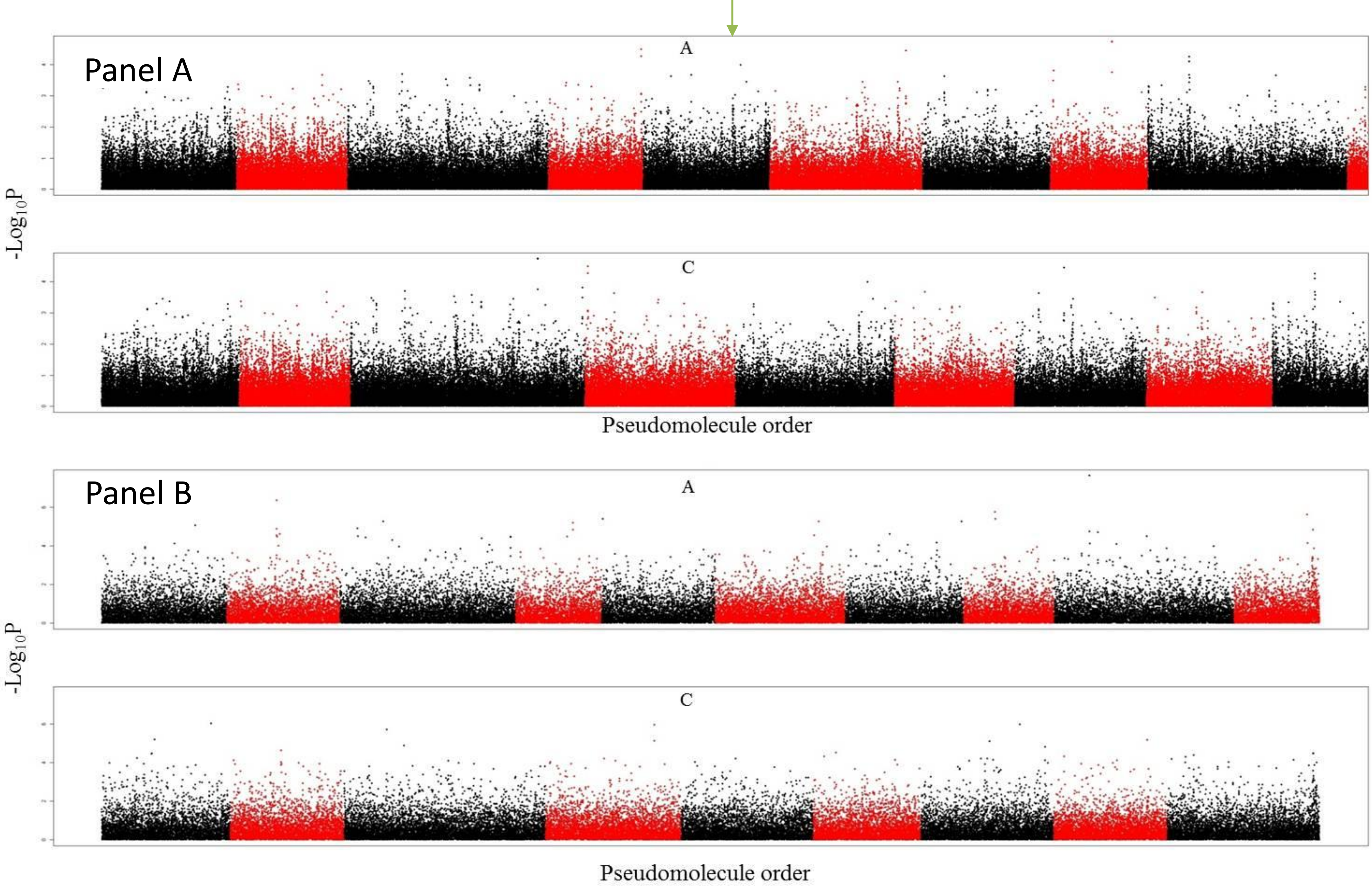

Supplementary Figure 4. Manhattan plots showing the Associative Transcriptomics SNP (A) and GEM (B) results for stem weight for JIC-grown *B. napus.* Significance of marker associations is shown as –Log10P and markers are plotted in pseudomolecule order. The two *B. napus* genomes can be seen marked as A and C.

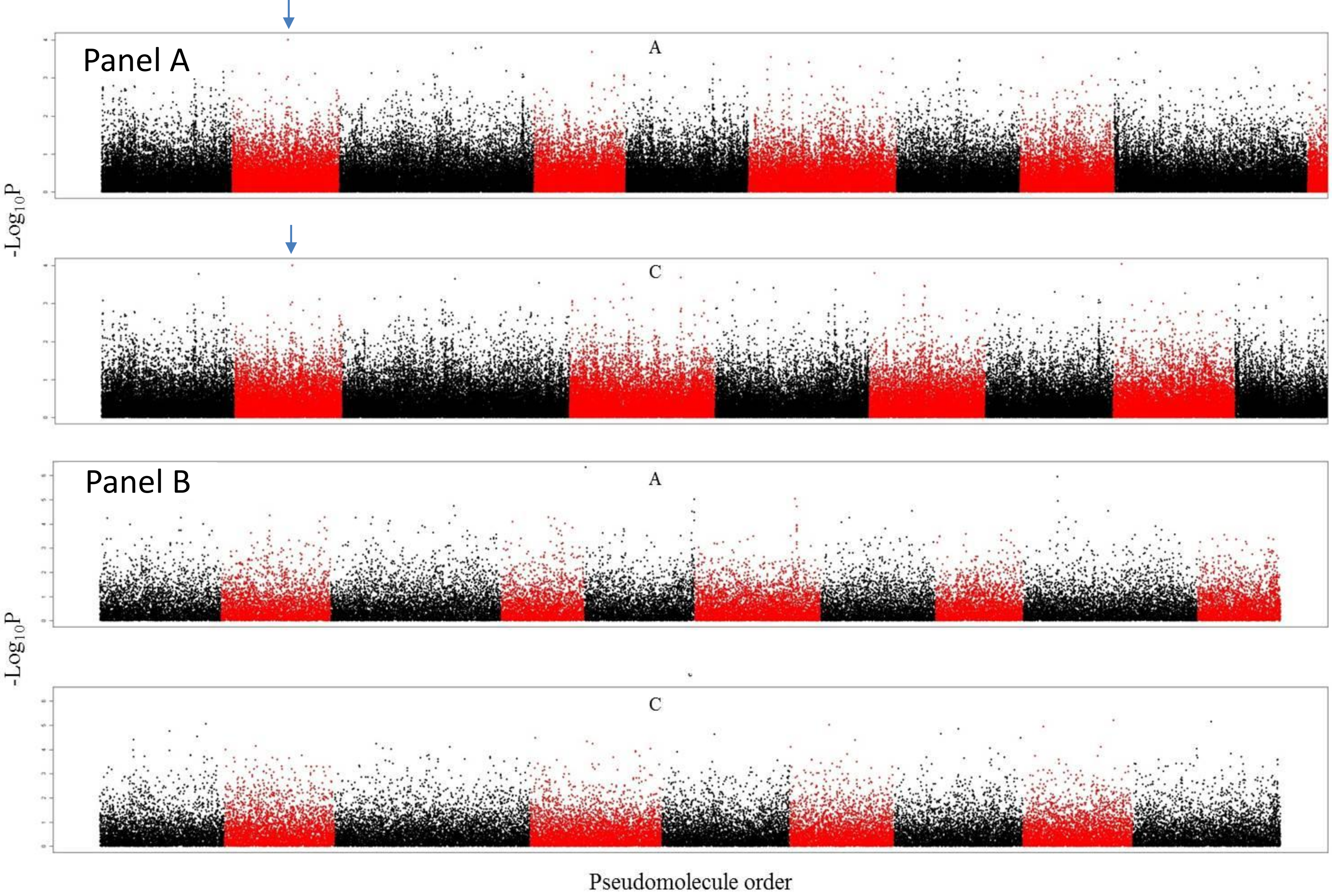

Supplementary Figure 5. Manhattan plots showing the Associative Transcriptomics SNP (A) and GEM (B) results for stem diameter for JIC-grown *B. napus.* Significance of marker associations is shown as –Log10P and markers are plotted in pseudomolecule order. The two *B. napus* genomes can be seen marked as A and C.

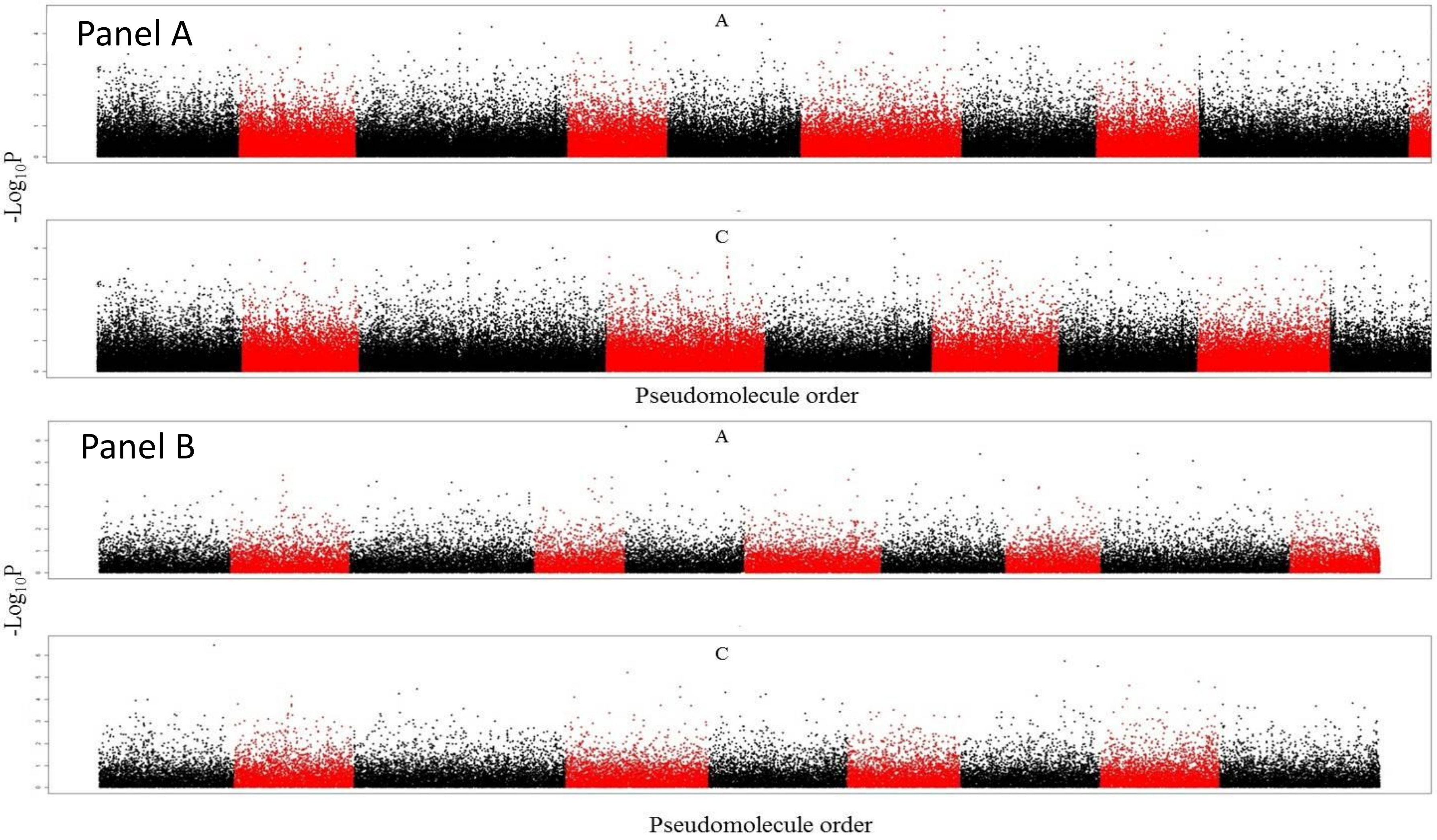

Supplementary Figure 6. Manhattan plots showing the Associative Transcriptomics SNP (A) and GEM (B) results for second moment of area for JIC-grown *B. napus.* Significance of marker associations is shown as –Log10P and markers are plotted in pseudomolecule order. The two *B. napus* genomes can be seen marked as A and C

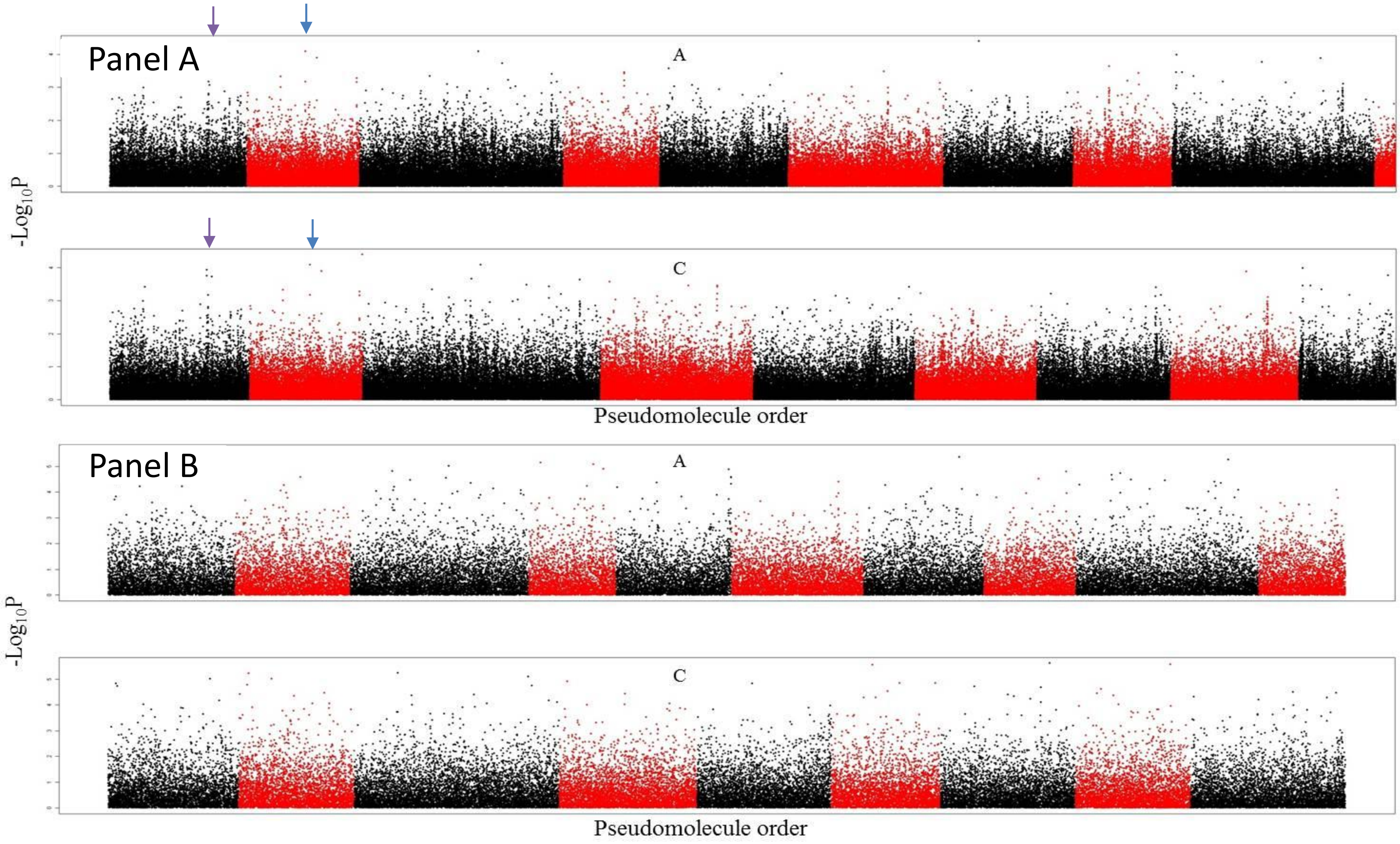

Supplementary Figure 7. Manhattan plots showing the Associative Transcriptomics SNP (A) and GEM (B) results for stem parenchyma area for JIC-grown *B. napus*. Significance of marker associations is shown as –Log10P and markers are plotted in pseudomolecule order. The two *B. napus* genomes can be seen marked as A and C.

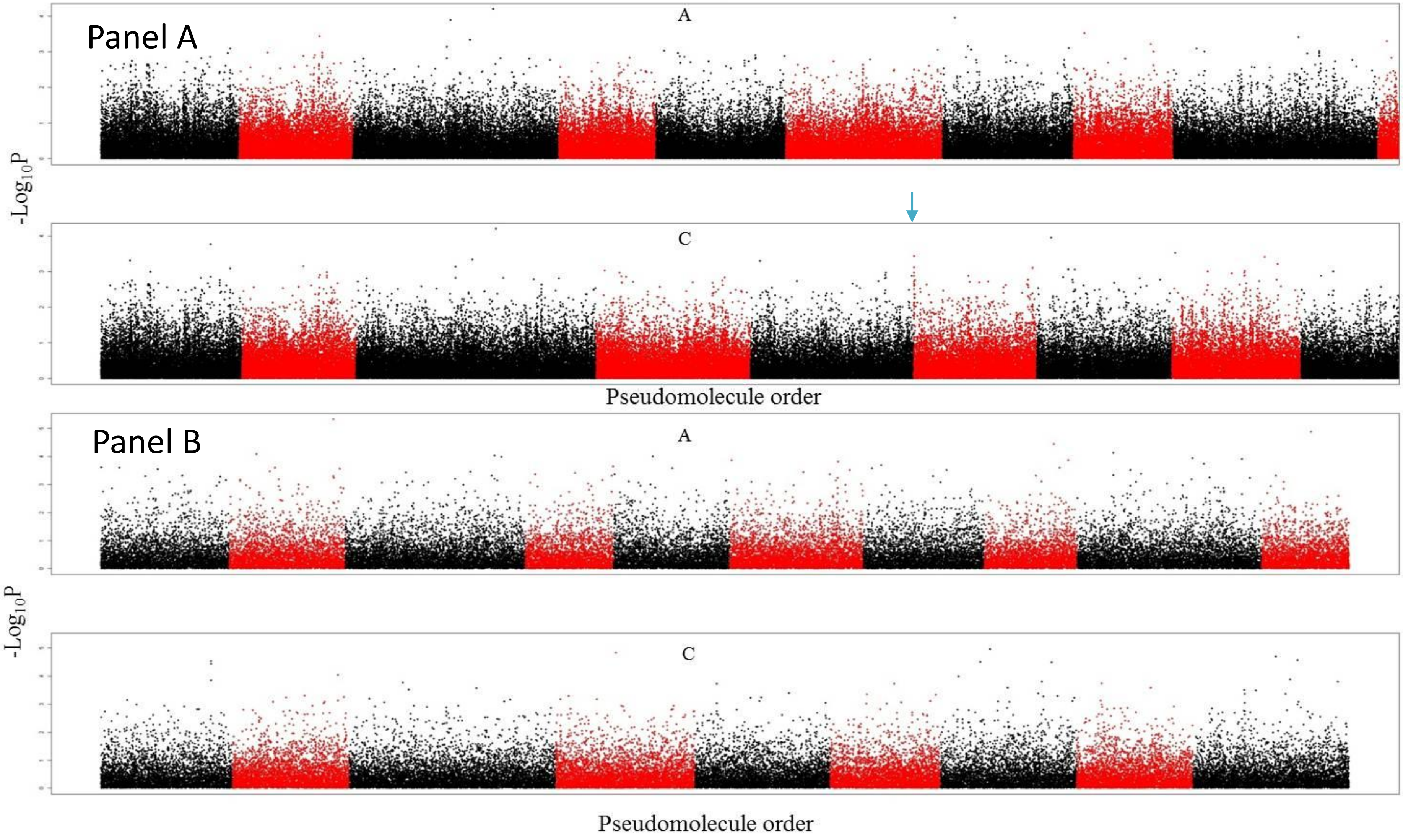

Supplementary Figure 8. Manhattan plots showing the Associative Transcriptomics SNP (A) and GEM (B) results for stem outer cortex thickness for JIC-grown *B. napus*. Significance of marker associations is shown as –Log10P and markers are plotted in pseudomolecule order. The two *B. napus* genomes can be seen marked as A and C.

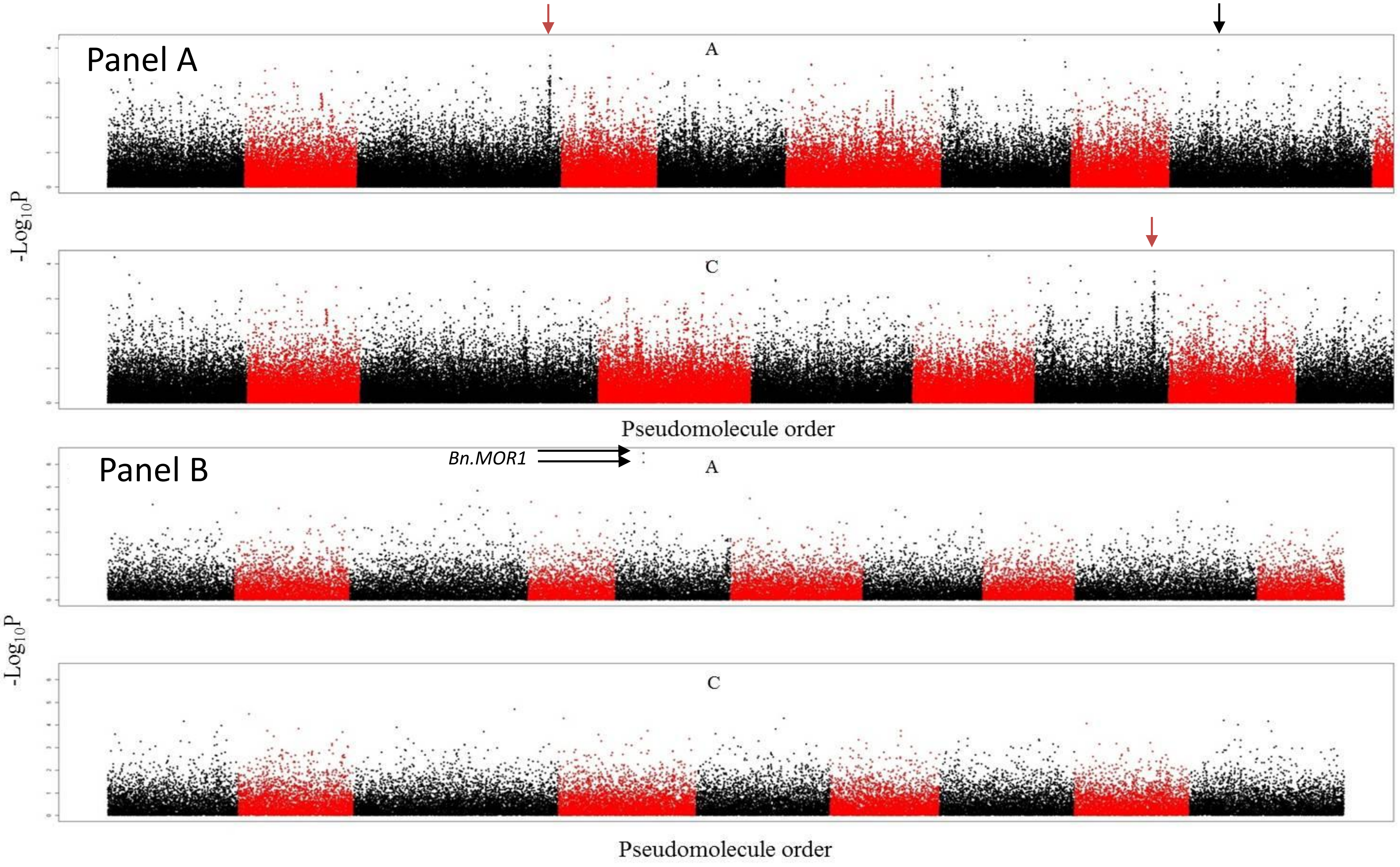

Supplementary Figure 9. Manhattan plots showing the Associative Transcriptomics SNP (A) and GEM (B) results for plant height for JIC-grown *B. napus*. Significance of marker associations is shown as –Log10P and markers are plotted in pseudomolecule order. The two *B. napus* genomes can be seen marked as A and C.

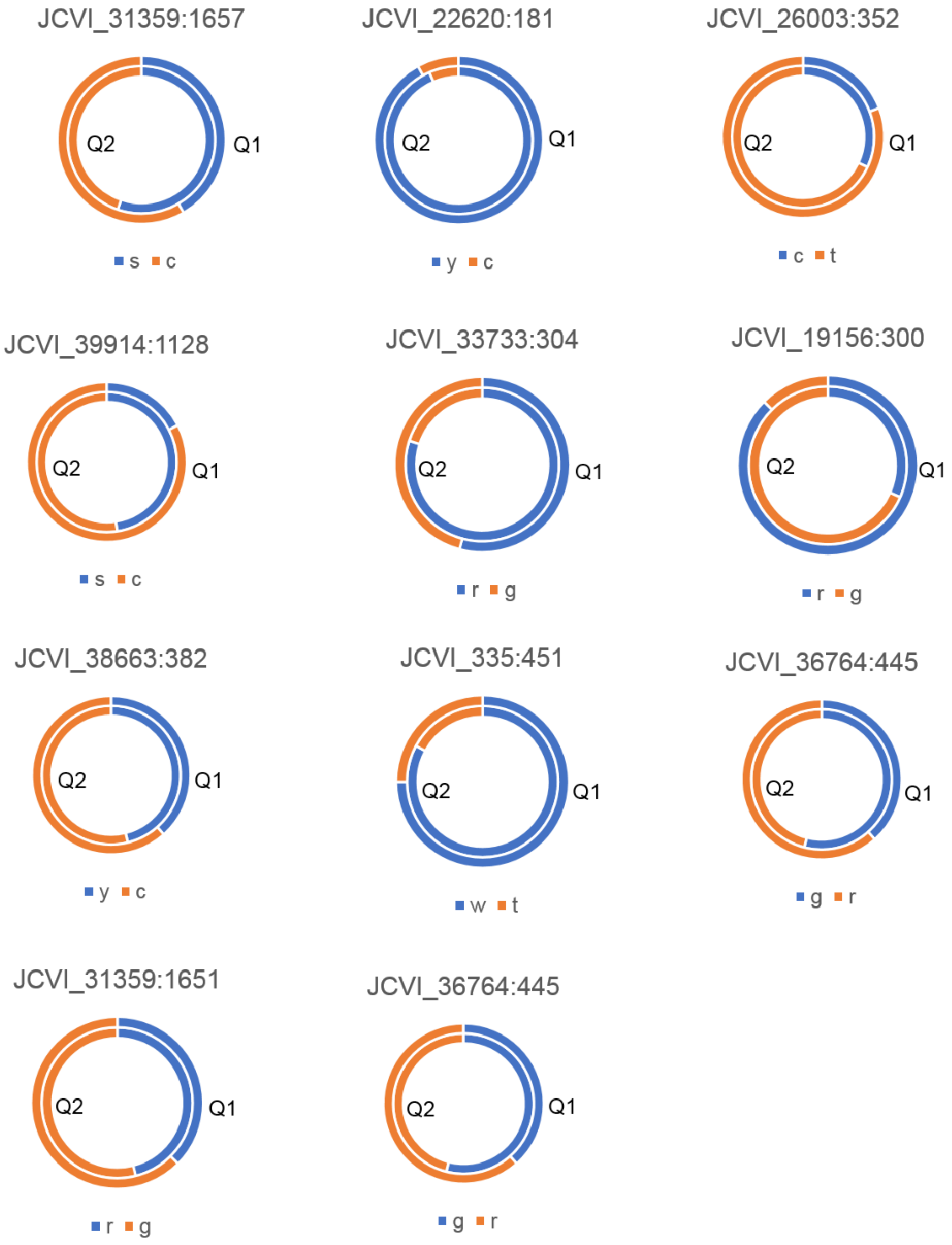

Supplementary Figure 10. Frequency in sub-populations of SNP markers associated with trait variation. The proportions of alleles of markers associated with trait variation in the sub-populations defined by STRUCTURE (Q1, Q2) are illustrated.
